# Editorial: Unlocking the power of gut microbiota to improving health and welfare in non-ruminant livestock

**DOI:** 10.3389/fvets.2025.1674586

**Published:** 2025-10-09

**Authors:** Shuai Liu, Yanting Chen, Jinghui Li, Mingyao Yang

**Affiliations:** ^1^State Key Laboratory of Animal Nutrition and Feeding, International Calf and Heifer Organization, College of Animal Science and Technology, China Agricultural University, Beijing, China; ^2^College of Animal Science and Technology, Nanjing Agricultural University, Nanjing, China; ^3^Department of Animal Science, The University of Chicago, Chicago, IL, United States; ^4^Center for Reproductive Biology, School of Molecular Biosciences, College of Veterinary Medicine, Washington State University, Pullman, WA, United States

**Keywords:** non-ruminant livestock, nutrient utilization, gut microbiota, intestinal health, animal welfare

## Introduction

In the era of sustainable agriculture, improving the health and welfare of non-ruminant livestock—including poultry, rabbits, and swine—has become a central focus for researchers and producers alike. Among the many factors that influence these outcomes, the gut microbiota emerges as a key player, orchestrating processes ranging from nutrient utilization to immune function. Recent studies, including those featured in this Research Topic, highlight how manipulating the gut microbiome through dietary strategies can unlock significant improvements in animal health, productivity, and welfare. This collection includes nine new studies that thoroughly explore these topics and other related subjects. The aim is to deepen our understanding of the importance of non-ruminant animals in global livestock production and to underscore the crucial role of gut microbiota in maintaining their health and welfare. The nine articles focus on aspects such as gut microbial communities, nutritional metabolism, health status, and production performance in non-ruminant species—including piglets, broiler chickens, rabbits, and horses. Emphasis is placed on the significance of dietary management strategies, such as functional additives and rearing systems, in promoting gut health and overall animal wellbeing.

## Gut microbiota: a driver of nutrient utilization

The microorganisms that reside in the gastrointestinal tract contribute significantly to the breakdown and absorption of nutrients. Non-ruminant livestock, including pigs and poultry, have different digestive systems compared to their ruminant counterparts. Their gut microbiota plays a crucial role in nutrient utilization. Non-ruminant livestock rely on their gut microbiota to break down complex nutrients that their own digestive enzymes cannot process, directly impacting feed efficiency, growth performance, and health condition (1). Research consistently demonstrates that microbial communities maintained within a stable environment can not only enhance the utilization efficiency of energy and amino acids in feed but also improve animal health and welfare (1–3). Jia et al. found in a 28-day clinical trial on weaned piglets that dietary supplementation with 5% and 10% fermented wheat bran significantly reduced the incidence of diarrhea and markedly improved the efficiency of nutrient absorption. These effects were associated with alterations in the gut microbiota, notably a substantial proliferation of beneficial bacteria such as *Prevotellaceae* and *Succinivibrionaceae*, which effectively promoted the digestion and absorption of dry matter, crude protein, and energy. Similarly, in broiler chicken production, the addition of fermented Astragalus polysaccharides (FAP) and glycyrrhiza extract containing Lactobacillus acidophilus (GUE) significantly enhanced nutrient utilization (Liu Z. et al.; Li X. et al.). Broilers supplemented with FAP not only exhibited higher daily weight gains but also produced carcasses with superior weights at slaughter (Liu Z. et al.). This improvement is attributed to an optimized microbial community structure that decomposes nutrients more efficiently. These findings suggest that precise modulation of microbial communities can optimize nutrient utilization, reduce waste, and enhance production efficiency.

## Safeguarding intestinal health through microbial balance

Intestinal health is a cornerstone of animal welfare, as a compromised gut barrier leads to inflammation, disease, and reduced productivity (4). The gut microbiota plays a critical role in maintaining this barrier by regulating tight junction proteins, inhibiting pathogens, and modulating immune responses (5). According to Liu Y. et al. weaned piglets fed a high-protein diet are at an increased risk of diarrhea; however, supplementation with Bacillus subtilis PB6 alleviated this Research Topic. This specific diet decreased diarrhea scores, enhanced growth performance, and modulated the gut microbiota, while also reducing the expression of inflammation-related genes. For broilers, Li X. et al. reported that in broiler chickens, a combination of *Glycyrrhiza uralensis extract* (GUE) and lactobacilli significantly increased the antioxidant enzyme levels in the intestinal mucosa, thereby reducing oxidative stress. Liu Z. et al. found that fermented *Astragalus* polysaccharides (FAP) also strengthened the intestinal barrier by upregulating tight junction proteins, which prevent pathogen entry. Wu et al. discovered that rearing systems influenced the ileal microbiota of rabbits. Forest-raised rabbits exhibit microbiota associated with reduced inflammation, whereas caged rabbits show alterations in metabolic pathways. These outcomes highlight the role of the microbiota as a “first line of defense” against intestinal dysfunction.

## Enhancing animal welfare: beyond productivity

Animal welfare encompasses more than just growth—it also includes freedom from disease, stress, and discomfort. The gut microbiota influences welfare through its impact on immune function, stress response, and even behavior (6). Ma et al. reported that in neonatal foals, angular limb deformities (ALDs) are linked to gut microbiota imbalances. Diseased foals and their mothers show altered microbial communities, which affect metabolism and immunity, highlighting the role of the microbiota in overall health. In broiler chickens, studies by Yan et al. and Li J. et al. demonstrated that dietary supplementation with adjuncts such as fructooligosaccharides (FOS) and vitamin D3 enhances meat quality and mitigates physiological stress. FOS-supplemented broilers were found to have better muscle pH and tenderness, while vitamin D3 modulated the cecal microbiota to enhance immune competence. Furthermore, Al-abdullatif et al. demonstrated that the administration of probiotics, including multi-strain formulations such as multi-strain probiotics (RISCO-NUTRIFOUR^®^, RNF), exerts beneficial effects on broiler welfare. Specifically, these probiotics enhance meat quality by decreasing cooking losses and increasing tenderness. Different concentrations of RNF have varying effects on the quality characteristics of the meat, such as texture, juiciness, and sensory acceptability, all of which contribute to the overall welfare perception in broilers, both during rearing and in the final product. These studies confirm that a healthy microbiome is integral to reducing suffering and improving quality of life.

## Harnessing microbial power: practical strategies

This Research Topic underscores actionable strategies to manipulate the gut microbiota to achieve better outcomes. Dietary additives (7)—including prebiotics (FOS), probiotics (Lactobacillus), plant extracts (GUE), and fermented polysaccharides (FAP)—consistently emerge as effective tools. For piglets, fermented wheat bran (FWB) and Bacillus subtilis PB6 are viable options. For poultry, a range of additives works: FAP for growth and antioxidant capacity, GUE-Lactobacillus combinations for immunity, and FOS for meat quality. Even rearing systems, such as forest-rearing for rabbits, can be leveraged. These strategies align with the push for antibiotic-free farming, as a balanced microbiota naturally suppresses pathogens.

## Conclusion

The gut microbiota plays a crucial role in the health and welfare of non-ruminant livestock, with far-reaching impacts on nutrient utilization, intestinal integrity, and overall wellbeing. This Research Topic demonstrates that targeted microbial modulation—through dietary additives and improved rearing systems—can unlock significant benefits, from enhanced productivity to reduced suffering. As the industry evolves, these insights will be key to raising healthier, more productive animals while meeting consumer demands for ethical and high-quality livestock products. Future research should focus on unraveling species-specific microbial interactions and refining interventions to maximize these gains for producers, animals, and consumers alike.
